# Berberine-Incorporated Shape Memory Fiber Applied as a Novel Surgical Suture

**DOI:** 10.3389/fphar.2019.01506

**Published:** 2020-01-09

**Authors:** Wen-cheng Zhou, Peng-fei Tan, Xing-han Chen, Ying Cen, Chao You, Lin Tan, Hao Li, Meng Tian

**Affiliations:** ^1^ Department of Burns and Plastic Surgery, West China Hospital, Sichuan University, Chengdu, China; ^2^ Neurosurgery Research Laboratory, West China Hospital, Sichuan University, Chengdu, China; ^3^ College of Biomass Science and Engineering, Sichuan University, Chengdu, China; ^4^ Key Laboratory of Leather Chemistry and Engineering of Ministry of Education, Sichuan University, Chengdu, China; ^5^ Department of Neurosurgery, West China Hospital, Sichuan University, Chengdu, China

**Keywords:** berberine, antibacterial activity, shape memory, surgical suture, wet spinning

## Abstract

The surgical suture has long been used to reconnect the injured tissues to restore their structure and function. However, its utility remains challenging in many areas, such as surgical site infections and minimally invasive surgeries. Herein, we report a novel surgical suture that possesses both antibacterial activity and shape memory effect to address these issues. In detail, natural antibacterial berberine was incorporated directly into the spinning solution of shape memory polyurethane with a near body transition temperature, and then berberine-containing polyurethane (BP) fibers were prepared by a facile one-step wet-spinning method for surgical suture. The prepared BP fibers were micro-sized and characterized by their transition temperature, morphology, water contact angles, mechanical properties, *in vitro* shape memory effect, drug release, and antibacterial activity. The results showed that with the increasing amount of the incorporated berberine, the transition temperatures of the fibers were not significantly affected, remains at near body temperature, while the contact angles of the fibers were significantly decreased and the mechanical properties of the fibers were significantly weakened. The optimized fiber was selected to evaluate the cytotoxicity and *in vivo* biocompatibility before *in vivo* shape memory effect and wound healing capacity in a mouse skin suture–wound model was tested. Besides the shape memory effect, it was demonstrated that the fiber is capable of antibacterial activity and anti-inflammatory effect, and promoting wound healing. The mechanism of the antibacterial activity and anti-inflammatory effect of the fiber was discussed. Overall, it is expected that by the berberine added to the fiber for surgical suture, it will be more popular and extend the utility of the sutures in a wide range of clinical applications.

## Introduction

As one of the most important medical devices, like wound dressing materials (Huang et al., 2019a), the surgical suture has long been used to reconnect the injured tissues to restore their structure and function (Shao et al., 2016). However, its utility remains challenging in many areas due to the increasing demands in the clinic. For example, surgical site infections (SSIs) are the second most common healthcare-associated infections that occur during the first 3 weeks postoperatively, resulting in prolonged hospital stays, readmissions, increased morbidity and mortality, and high costs (Joseph et al., 2017). Although the underlying causes have been suggested, such as bacteria bio-adherence and formation of colonization and subsequent biofilm in the surgical site, there is no effective treatment to improve the outcomes of the patients according to clinical feedback.

Antibacterial treatment of surgical suture is a promising strategy to address SSIs as it has shown the feasibility of inhibiting bacterial growth in wounds and reducing the infection rate, such as FDA-approved triclosan-impregnated Polyglactin 910 (Byrne and Aly, 2019). Nevertheless, the wide use of triclosan has been suggested to a potential selection of bacteria and triclosan-adapted cross-resistance with antibiotics. Therefore, new alternative substances need to be employed so as to achieve good antibacterial activity. In this regard, berberine hydrochloride (BCH), which is derived from *Coptis chinensis*, was applied in this study. As a natural active pharmaceutical ingredient, it has gained considerable attention for its manifold pharmacological properties, including antibacterial activity and anti-inflammatory effect, both of which have been suggested to be beneficial to the infected wound, indicating that berberine might be a promising candidate for the treatment of surgical suture (Avila-Carrasco et al., 2019).

For the fabrication of antibacterial surgical suture, there are mainly two approaches that have been involved. The drug-coating approach has its intrinsic advantages of easy production at low cost without compromising the mechanical property of the suture matrix, whereas the major drawback lies in low drug loading and poor control over the drug release. On the contrary, the drug incorporation method such as electro-spinning is beneficial to attaining high efficient drug loading, while the production process generally needs harsh post-treatment that would inactivate the loaded drugs (Feng et al., 2019; Ding et al., 2019). For instance, commercial sutures have been commonly fabricated by melt spinning; however, the use of high temperature during the processing steps would be harmful to the activities of the loaded drugs.

Besides SSIs, the surgical suture was also challenged in difficulty in handling in minimally invasive surgery, in which knotting a suture was limited in a confined space. In this case, if the knot was fixed with a force that is too weak, scar tissue would form, leading to the formation of hernias and bacteria invasion. Instead, when the force was too strong, necrosis of the surrounding tissue may occur, resulting in patients suffering from severe pain after the surgery. One solution to overcome this obstacle would be to use a smart suture with shape memory effect triggered by the body temperature, e.g., the suture was elongated to be applied loosely in its temporary shape first, and then would shrink and tighten the knot in an adapted force at body temperature, and thus suture with shape memory effect seems to be an effective approach to improving the handling in surgery.

**Figure 1:**
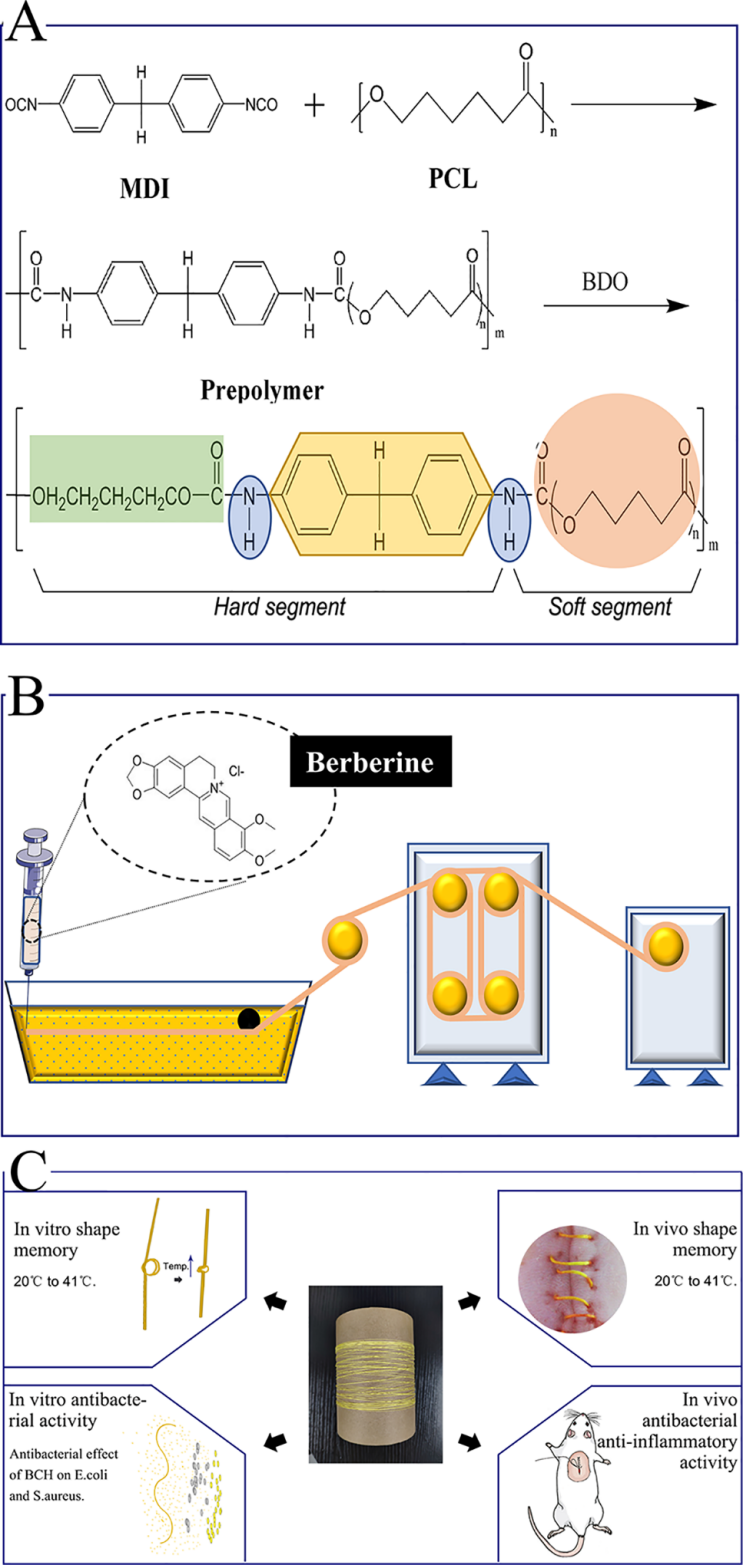
Schematic presentation of the whole experiment. **(A)** Synthesis of the SMPU; **(B)** The one-step wet-spinning method for fiber; **(C)** *In vitro* and *in vivo* assays.

Herein, we report a novel surgical suture possessing both antibacterial activity and shape memory effect to address these issues. Specifically, natural antibacterial berberine was incorporated directly into the spinning solution of a shape memory polyurethane with a near body transition temperature that synthesized as our previous study (the synthesis route is shown in **Figure 1A**), and then berberine-containing polyurethane (BP) fibers were prepared by a one-step wet-spinning method for surgical suture (**Figure 1B**). The prepared BP fibers were characterized by their transition temperature, morphology, water contact angles, mechanical properties, *in vitro* shape memory effect, drug release, and antibacterial activity. The optimized fiber was selected to evaluate the cytotoxicity and *in vivo* biocompatibility before *in vivo* shape memory effect and wound healing capacity in a mouse skin suture–wound model was tested (**Figure 1C**). The mechanism of antibacterial activity and anti-inflammatory effect of the BP fiber was proposed and discussed.

## Materials and Methods

### Materials

Polycaprolactone diol (PCL, Mn = 4000 g/mol) was purchased from Perstop UK Ltd. (United Kingdom). Berberine hydrochloride (BCH, 98% purity) was supplied by Aladdin Co., Ltd. (Shanghai, China). *N*,*N*-dimethylacetamide (DMAc) and other solvents were obtained from the Chengdu Kelong Reagent Company and used directly without further purification. *Escherichia coli* (ATCC8739) and *Staphylococcus aureus* (ATCC6538) were supplied by the R&D Lab of Functional Fibers of Sichuan University, China. Hematoxylin and eosin (H&E) stains were purchased from Baso Diagnostics Inc. (China). Gram’s crystal violet solution (G1060) was purchased from Beijing Solarbio Science & Technology Co., Ltd. Two antibodies, interleukins (interleukin-1beta, IL-1β) and tumor necrosis factor (TNF-α), were purchased from Santa Cruz Biotechnology, Inc. (USA). Alamar Blue was purchased from Beijing Cell Chip Biotechnology Co., Ltd. (Beijing, China).

### Synthesis of SMPU

Shape memory polyurethane (SMPU) was synthesized according to our previous study (Tang et al., 2019). The synthesis route is shown in **Figure 1A**. For the obtained SMPU, the thermal transition temperature (*T*_trans_) based on the melting temperature (*T*_m_) of soft segments PCL was a near body transition temperature around 41°C.

### Preparation of Berberine-Containing Shape Memory Fiber for Surgical Suture

The synthesized SMPU was first softened at 60°C and then cut into small chips. A certain amount of SMPU chips were dissolved in DMAc under 60°C within 6 h to obtain a homogenous solution. Twenty percent mass concentration of SMPU was applied as the primary spinning solution, which was named SS-0. Similarly, two composite solutions were prepared, which were named SS-1 and SS-2 according to spinning solutions of 20%SMPU + 3%BCH and 20%SMPU + 6%BCH, respectively. **Table S1** summarizes the spinning parameters for preparing the fibers.

The prepared spinning solution was loaded into a syringe. The internal diameter of the syringe needle was 0.84 mm. During the process of spinning, the spinning solution was extruded from the syringe and solidified in the coagulation bath containing calculated pure water, and then the fibers passed through the drawing roller, drying rollers, and finally collected on a rotating mandrel. Three fibers were successfully obtained and named as BP-0, BP-1, and BP-2 based on the spinning solutions of SS-0, SS-1, and SS-2, respectively.

In the process of spinning, the amount of spinning solution was calculated by weighing, and the absorbance value of the coagulation bath after spinning was measured by an ultraviolet spectrophotometer. The content of berberine leaked into the coagulation bath was calculated according to the standard curve of berberine so as to calculate the actual drug load of berberine in the fiber.

### Characterization of the Fibers

The surface and cross-section morphologies of the fibers were observed under scanning electron microscopy (SEM) (SU3500, Hitachi, Japan) after gold spraying. The static water contact angles of the fibers were tested with a surface contact angle tester (Harke-SPCAX1, China). Each sample was tested three times in parallel, and the average value was used as the final water contact angle to reflect the hydrophilicity and hydrophobicity of the fiber. The mechanical properties of the fibers were tested with an electronic single-yarn strength machine (YM061, China), in which the clamping distance was 50 mm, the tensile rate was 100 mm/min, and the fracture threshold was 85%. Differential scanning calorimetry (DSC) was performed on a DSC-Q200 instrument (Hitachi, Japan).

### *In Vitro* Shape Memory Test

Shape memory performance was observed by stretching the original length of 6 cm fiber to 12 cm above its *T*_trans_ and fixing with deionized water at room temperature, and then heating the fibers again. The fixed ratio (*R*_f_) and recovery rate (*R*_r_) of the fibers were calculated using the following equations:

$$R_{f}=(L_{2}-L_{0})/(L_{1}-L_{0})\times 100\%$$

$$R_{r}=(L_{1}-L_{3})/(L_{1}-L_{0})\times 100\%$$

where L_0_, L_1_, L_2_, and L_3_ stand for the lengths of the initial shape, deformed shape, fixed shape, and recovery shape, respectively.

### *In Vitro* Drug Release

Prior to the antibacterial and animal experiments, we investigated the release behaviors of berberine from the normal fibers and stretched ones to confirm that berberine can release from the fibers and then exhibit the antibacterial performance. In virtue of the shape memory function, the fibers can be deformed under higher temperatures and maintain the deformed shapes under lower temperatures. BP-1 and BP-2 fibers were stretched by 100% before the berberine release, and the deformed shapes were fixed at room temperature, which were named BP-1S and BP-2S, respectively. BP-1, BP-2, BP-1S, and BP-2S fibers in triplicate were put into 5-ml phosphate-buffered saline (PBS) buffer solution with different pH values, including 4.0, 7.0, and 9.0, respectively, and placed in a shaker with a constant temperature of 37°C. Three milliliters buffer solution was withdrawn at several regular intervals and an equal amount of PBS was supplemented until the release was completed. The released amount of berberine was determined by a UV–visible spectrophotometer at 344 nm, and the accumulated release curves were plotted according to the standard curve.

### Antibacterial Test

Two representative strains of *E. coli* and *S. aureus* were selected to demonstrate the antibacterial performance of the prepared fibers embedded with berberine. The test procedure was conducted as follows: 1) The bacterial strain was incubated in Luria–Bertani (LB) broth at 37°C for 12 h, and the original bacterial suspension concentration was diluted to 1 × 10^5^–5 × 10^5^ CFU/ml with PBS buffer with pH 7.0; 2) 20 mg BP-0, BP-1, and BP-2 fibers were individually placed inside testing vials containing 1.5 ml PBS, and then adding 1.0 ml of diluted bacterial suspension into the vials for another 12 h incubation; 3) the co-cultured bacterial suspension was diluted into several concentrations to determine the quantity of bacteria, and 100 μl of the bacterial suspension was withdrawn to spread on the LB agar plates and subsequently incubated at 37°C overnight; (4) observing and counting the colonies on LB agar plates. The bacterial suspensions without fiber were used as blank controls.

### *In Vitro* Cytotoxicity

*In vitro* cytotoxicity of the fibers was assayed according to ISO 10993 part 5 guidelines 3 (Bernard et al., 2017). Extract was prepared by the incubation of 0.2 g of each fiber with 1 ml of Dulbecco’s modified Eagle’s medium (DMEM) supplemented with 10% fetal bovine serum (FBS), 100 μg/ml penicillin, and 100 μg/ml streptomycin for 48 h at 37°C. L929 fibroblasts were cultured until they reached approximately 80% confluency before preparing the plates for the cytotoxicity assay. The density of seeding was 5,000 cells per well in the 96-well plates. After 24 h, the culture media were discarded and replaced with the extracts. Alamar Blue assay was performed for the cytotoxicity test in accordance with the manufacturer’s instruments (Mullick Chowdhury et al., 2013). Briefly, the cells were washed with PBS before adding 100 μl phenol red-free DMEM and 10 μl of Alamar Blue reagent (Bernard et al., 2017). After 4 h incubation, fluorescence measurements were recorded using a Multifunctional Fluorescent Enzyme Marker (BioTek Synergy Mx, USA) at the respective excitation and emission wavelengths of 530 and 580 nm. Six parallels were carried out in each group. All viability values were calculated relative to the blank control.

### Animal Studies

#### Animals

The animal studies were approved by the Animal Ethical Committee of the West China Hospital of Sichuan University in compliance with Chinese national guidelines for the care and use of laboratory animals. Adult male BALB/C mice (25–30 g) were purchased from Dashuo Laboratory Animal Co., Ltd. (Chengdu, China). The mice were kept under controlled temperature and humidity with food and water *ad libitum*.

#### *In Vivo* Biocompatibility

The mice were anesthetized with intraperitoneal administration of pentobarbital sodium (3% in saline solution) at a dose of 1 ml/kg. The gluteal side of the mice was shaved and disinfected with iodine solution before surgery. The BP-1 suture, 3 cm long, was implanted into the left side of the gluteal muscle. To avoid secession of the suture construction, both ends of the suture were tied into a knot with a little space. Treatment with BP-0 suture in the right gluteal muscle was employed as control (Shao et al., 2016). After 3 and 7 days of operation, three mice in each group were sacrificed, respectively. H&E staining was performed to investigate the biocompatibility.

#### *In Vivo* Shape Memory

The *in vivo* shape memory test was performed as in the previous study, with minor modifications (Lendlein and Langer, 2002). Briefly, the sutures were first stretched twofold of their initial length and maintained for 10 min at 50°C, then cooled at room temperature and kept in ambient temperature before the following experiments. Three mice were anesthetized as before and 2-cm-long incisions were made on the back. Then the wound was sutured loosely. The shape memory effect was actuated while the temperature of antecedent trauma was increased from 20°C to 37°C, and to 41°C by a hair blower to simulate a posttraumatic hyperpyrexia process. The photos of the same wound at different temperatures were shot from vertical and lateral directions.

#### Mouse Skin Suture–Wound Model

The sutures were first stretched twofold of their initial length and maintained for 10 min at 50°C, then cooled at room temperature and kept in ambient temperature before use. The mouse skin suture–wound model was established as described previously, with minor modifications (Mebert et al., 2018). Briefly, the mice were anesthetized with intraperitoneal administration of pentobarbital sodium (3% in saline solution) at a dose of 1 ml/kg. The fur of the test animals was shaved carefully using an electric razor and further removed completely using a depilatory cream. Then the skin was decontaminated with alcohol and iodophor solution. A 1.5-cm-length incision was made along the mid-back of the trunk. The depth of the wound did not break into the panniculus carnosus. One wound was created per animal. The sutures were threaded onto surgical needles and the continuous suture was chosen for the closure of the wounds (Wang et al., 2018). The mice were randomly divided into four groups as follows: G I (the wound was sutured with BP-0); G II (the wound was sutured with BP-1); G III (the wound was sutured with BP-0 before bacterial inoculation); and G IV (the wound was sutured with BP-1 before bacterial inoculation). For G III and IV, *S. aureus* was inoculated by applying 10 μl of bacterial suspension (1 × 10^8^) onto the incision site for infection (Nakagawa et al., 2017). For G I and II, the same volume (10 μl) of saline was applied as the control (Hahn et al., 2009). The wounds were photographed with a camera (Canon-IXUS230HS, Japan) and infrared thermal imaging (FLIR-T62101, Sweden) was taken every day.

For histology and immunohistochemistry, mice were euthanized after 3 and 7 days post-surgery and the skin tissues were harvested from the suture sites. The tissues were then fixed in 4% paraformaldehyde, dehydrated in graded alcohol, and embedded in paraffin. The embedded tissue blocks were sectioned at 5 μm thickness and stained with H&E, TNF-α, and IL-1β immunohistochemical staining. To identify bacteria, biopsy specimens were stained with Gram’s crystal violet solution. Images of the histological section were acquired using an Olympus compound microscope (BX43, Japan) connected with an Olympus microscope camera (Sence, Japan). In order to evaluate the severity of inflammation, ImageJ software was used for image analysis and quantitative counting (Fan et al., 2018). Four random high-power fields (HPFs) were chosen from each section. Neutrophils in 10 regions of interest (ROIs) per HPF were counted. There are four sections in total randomly chosen from a mouse.

### Statistical Analysis

The statistical methods were chosen according to the type of data. Two groups of unrelated data, if the variance is homogeneous, then unpaired *t* test was used. Comparisons between the multiple groups were determined by one-way ANOVA. Kruskal–Wallis was used for analyzing thermal recordings. All statistical calculations were carried out with the help of GraphPad Prism 7.0. Significance was defined as: *** for *P* < 0.001, ** for *P* < 0.01, and * for *P* < 0.05.

## Results and Discussion

### Characterization of the Fiber for Surgical Suture

**Figure 2:**
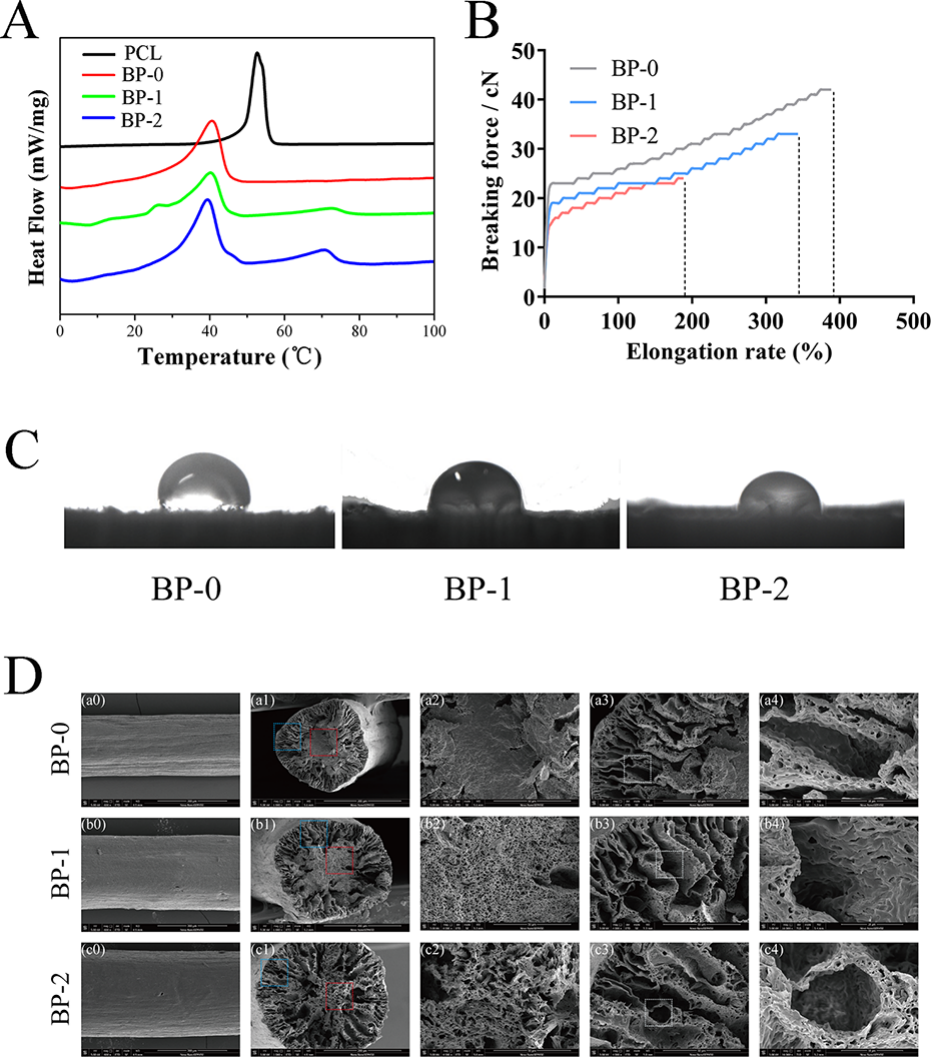
Typical DSC curves **(A)**, stress–strain curves **(B)**, water contact angles **(C)**, and SEM images **(D)** of the fibers.

The berberine-containing shape memory fiber was fabricated by a facile scalable one-step wet-spinning approach, and the fibers were micro-sized in the range of 237–339 μm. The fibers were characterized in terms of *T*_m_, drug loading, surface wettability, and mechanical properties, and the testing results are summarized in **Table 1** and **Figure 2**. According to the DSC curves (**Figure 2A**), the influence on the *T*_m_ after incorporating berberine is not obvious, and the prepared three types of fibers, BP-0, BP-1, and BP-2, have similar *T*_m_ which are near body temperature. Except that the *T*_m_ of pristine PCL exhibited the highest around 53°C, while that of other fibers was around 40°C, which was derived from the partial crystallization of PCL segments. In addition, with the increase of the incorporated berberine, the *T*_m_ of the fiber was decreased, indicating that the incorporated berberine also decreased the *T*_m_ of the fiber.

**Table 1:** Properties of the fibers (mean ± SD, *n* = 6).

Fiber	Diameter (μm)	*T*_m_ (°C)	Drug loading (%)	Contact angle (deg)	Breaking force (cN)	Elongation at break (%)
BP-0	237.35 ± 4.71	40.45	0	106.5 ± 0.3	42.10 ± 2.02	375.97 ± 13.68
BP-1	291.25 ± 5.09	40.36	12.70	93.3 ± 0.6	31.20 ± 2.35	287.47 ± 23.29
BP-2	339.38 ± 2.96	39.55	22.40	87.2 ± 0.8	24.40 ± 1.17	175.29 ± 7.57

**Figure 2B** shows the typical tensile curves of BP-0, BP-1, and BP-2 fibers. Clearly, the content of berberine in the fiber has a significant effect on the mechanical property of the fiber, i.e., with the increase in the amount of berberine in the fibers, both breaking strength and elongation decreased, which could be ascribed to the disruption effect of berberine; in other words, the integrity of the fibers were damaged by BCH particles, and more defects were yielded when increasing the amount of BCH.

The water contact angles (WCA) of the prepared fibers were measured using a sessile drop method. As shown in **Figure 2C** and **Table 1**, the WCA of the fibers was decreased with the increase of the incorporated berberine, which was resulted from the hydrophilic nature of berberine with quaternary ammonium structure. The more berberine incorporated into the fiber, the higher the hydrophilicity of the fibers, and thus the WCA value is in the order of BP-2 < BP-1 < BP-0.

The microstructure of the fibers was observed by SEM measurements. As shown in **Figure 2D**, the surface of the obtained fibers is almost smooth, and some shallow grooves can be found. More obviously, with the increase of the incorporated berberine, the core part of the fiber became looser and larger micropores were yielded, indicating that the distribution of berberine might also influence the fiber solidification and formation during the spinning process. Additionally, it could be inferred that a loose microstructure might lead to the reduction of mechanical property.

### *In Vitro* Shape Memory

**Figure 3:**
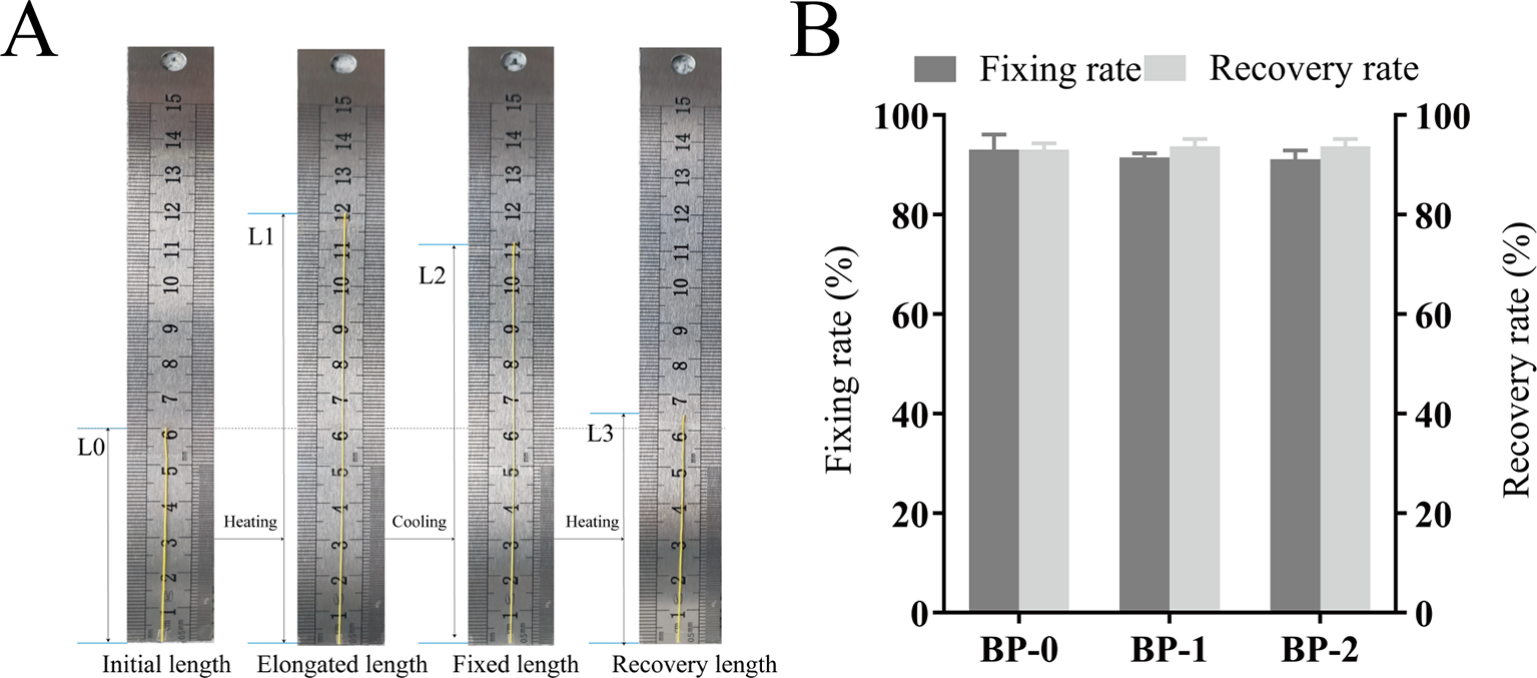
Macroscopic pictures for demonstrating the shape memory process **(A)** and the fixation and recovery ratios of different fibers **(B)** (mean ± SD, *n* = 6).

Prior to the *in vivo* application as the surgical suture, the shape memory performance was tested *in vitro*, and the testing process and the corresponding fixation and recovery ratios are shown in **Figure 3**. The whole shape memory process mainly includes four states in original shape (a), in maximum strain (b), in fixation (c), and after recovery (d), in which the fixed shape with longer length can be applied as the surgical suture, and then recover to the shorter length upon the trigger under body temperature (Lendlein and Langer, 2002). According to testing results, all the samples showed desirable *R*_f_ and *R*_r_, which were around 90% without significant difference, and such shape memory capacity can meet the requirement for tightening wounds in practical application. More specifically, the shape memory suture with elongated length can be easily applied on the wound in a pleasant way, and then shrink and recover to the original length gradually triggered by body temperature in virtue of the thermal sensitivity.

### *In Vitro* Drug Release

**Figure 4:**
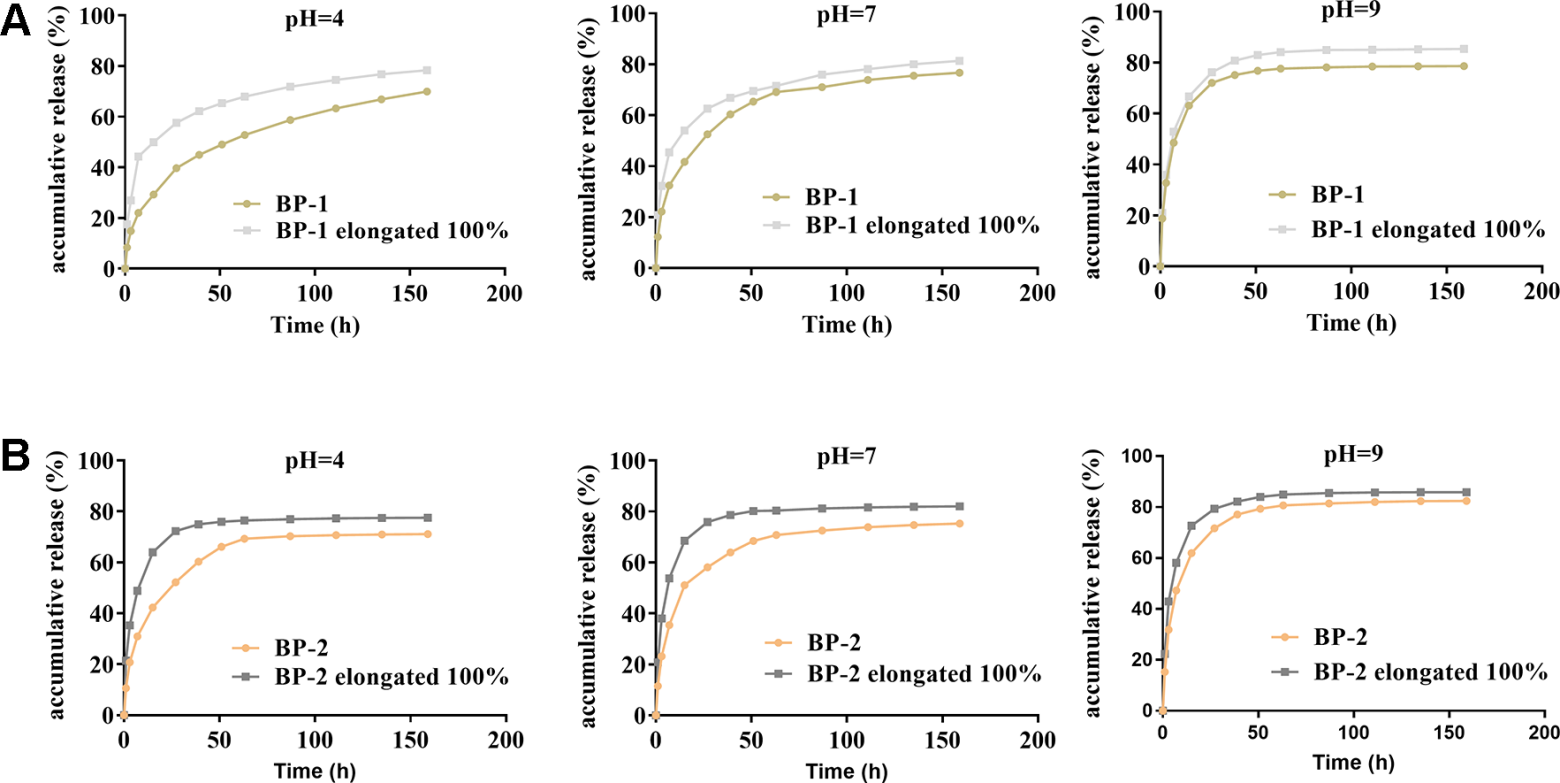
Effects of pH and stretching on the BCH release of the fiber BP-1 (**A**) and BP-2 (**B**) (mean ± SD, *n* = 6).

The *in situ* environment of the wound is commonly acidic due to inflammation or infection, and thus the effect of pH on the release of berberine was studied. As shown in **Figure 4**, the release profile of all fibers exhibited two stages. The first stage would be one of the rapid burst releases and the second would be one of slow and extension releases. The release of berberine for all fibers was significantly affected by pH values, i.e., the release of berberine was in the order of medium at pH 4 < pH 7 < pH 9. Considering the acidic environment of the wound, the slower release of berberine probably has the advantage of release in a sustained manner.

When shape memory fiber was used as suture, the fiber was stretched, which might accelerate the release due to the increased surface area and the reduced release pathway after stretching deformation. As expected, our results showed that berberine released faster from the fiber under stretched state compared to that from original fiber, which might be beneficial to early antibacterial actions that need a high dose of berberine to kill the bacteria.

### Antibacterial Activity

**Figure 5:**
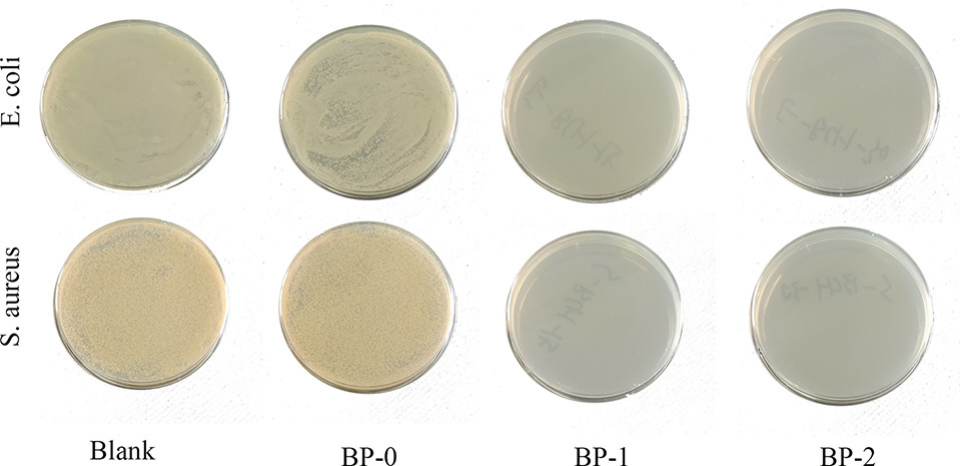
Colony growth of the fibers against *Escherichia coli* and *Staphylococcus aureus*.

The antibacterial performance of berberine was tested and the results are shown in **Figure 5** and **Table S2**. Obviously, bacteria spread fully on the agar plates of BP-0 and blank control groups, and the number of bacteria colonies derived from both groups were around 10^6^ CFU/ml. In contrast, few colonies can be found on the agar plates of BP-1 and BP-2 groups, indicating BP-1 and BP-2 have completely inactivated both *E. coli* and *S. aureus*.

### Cytotoxicity and *In Vivo* Biocompatibility

**Figure 6:**
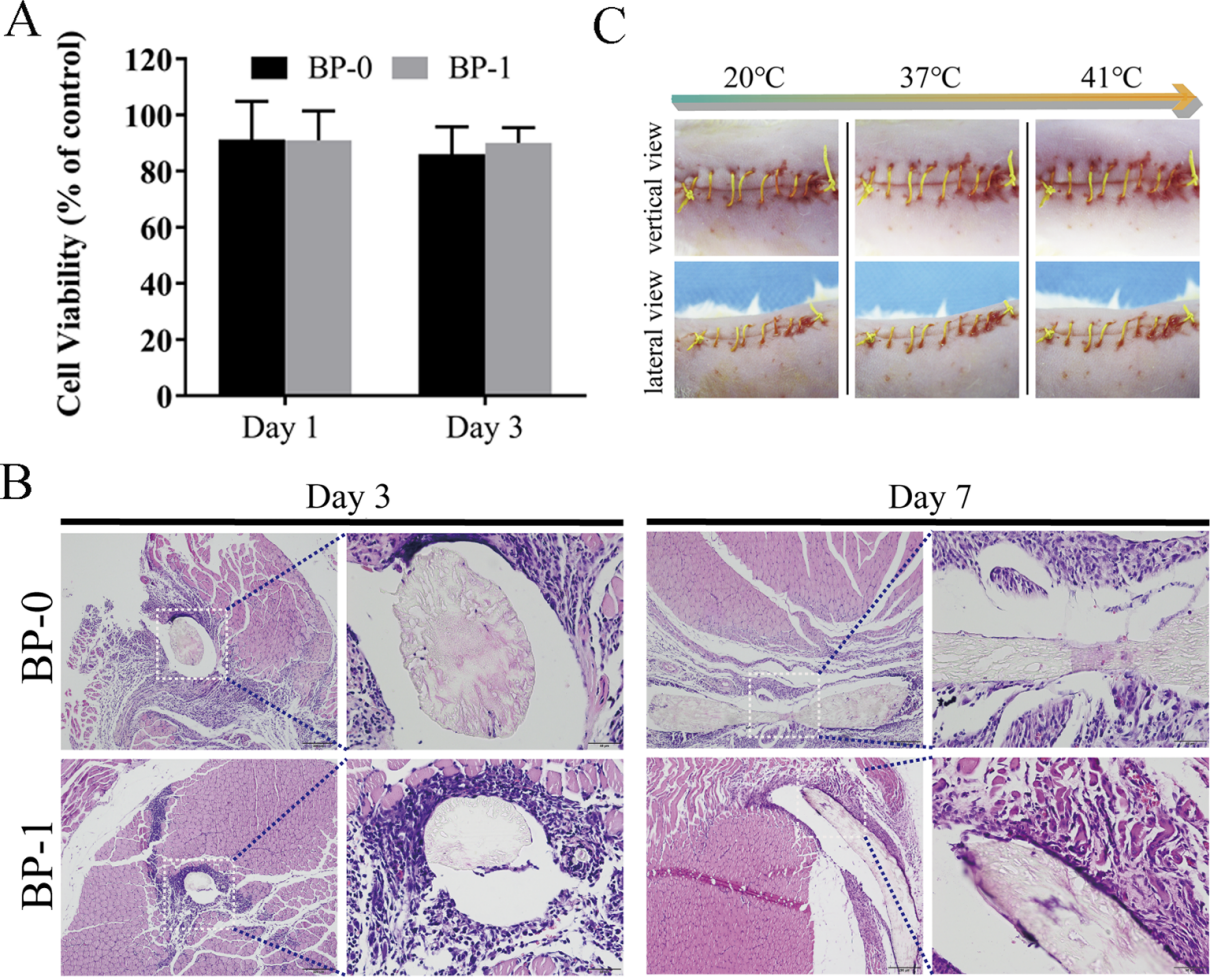
**(A)** Cytotoxicity test on days 1 and 3. Data are presented as the mean ± SD (*n* = 6). **(B)** *In vivo* muscular biocompatibility evaluation of sutures. Representative photos of H&E-stained histological sections of the muscle tissue with BP-0 and BP-1 after implantation for 3 and 7 days. Images on the *right* were the magnified ones from the *left dotted line marked ones*. **(C)** *In vivo* shape memory assay. The photo series (20°C to 41°C) shows the shrinkage of the fiber while temperature increases (vertical and lateral views for the same temperature).

Considering the mechanical property of the prepared fibers, BP-1 was selected as the optimized fiber for the following evaluation studies, and BP-0 was used as the control. The cytotoxicity of the BP-0 and BP-1 fibers was tested by Alamar blue assay using L929 cells. It is evident that after incubation for 1 and 3 days, the cell viability of the two fibers was around 90% (**Figure 6A**), indicating that there is almost no cytotoxicity for two sutures. The *in vivo* biocompatibility of the fibers was assayed by intramuscular implantation. As shown in **Figure 6B**, on day 3, both two fibers are surrounded by a large number of inflammatory cells, which evident a severe inflammation reaction at an early time. However, when the time prolonged to day 7, the inflammation reaction for BP-1 almost disappeared, which might be due to the pharmacological effects of berberine on anti-inflammation. In contrast, for BP-0, there was still a massive quantity of inflammatory cells around the fiber.

### *In Vivo* Shape Memory

As shown in **Figure 6C**, the elongated fiber is sutured loosely in the wound and it is, as expected, gradually shrinking when the temperature rises, indicating that the *in vivo* shape memory effect of the fiber was evident. It is uneasy for a doctor to handle the tying force by using traditional surgical suture. If the force of tying is too high, it may cause necroses of the surrounding tissue. On the contrary, if the tying force is too weak, it may result in scars and even non-union. Therefore, the shape memory effect of this novel suture provides a potential therapeutic strategy that doctors could suture wounds in its slack elongated state, and then the suture would be triggered by body temperature to return to its original shape and the contraction of suture makes the knot tight in an adapted force (Zhao et al., 2019).

### Mouse Skin Suture–Wound Model

#### Wound Healing Evaluation

**Figure 7:**
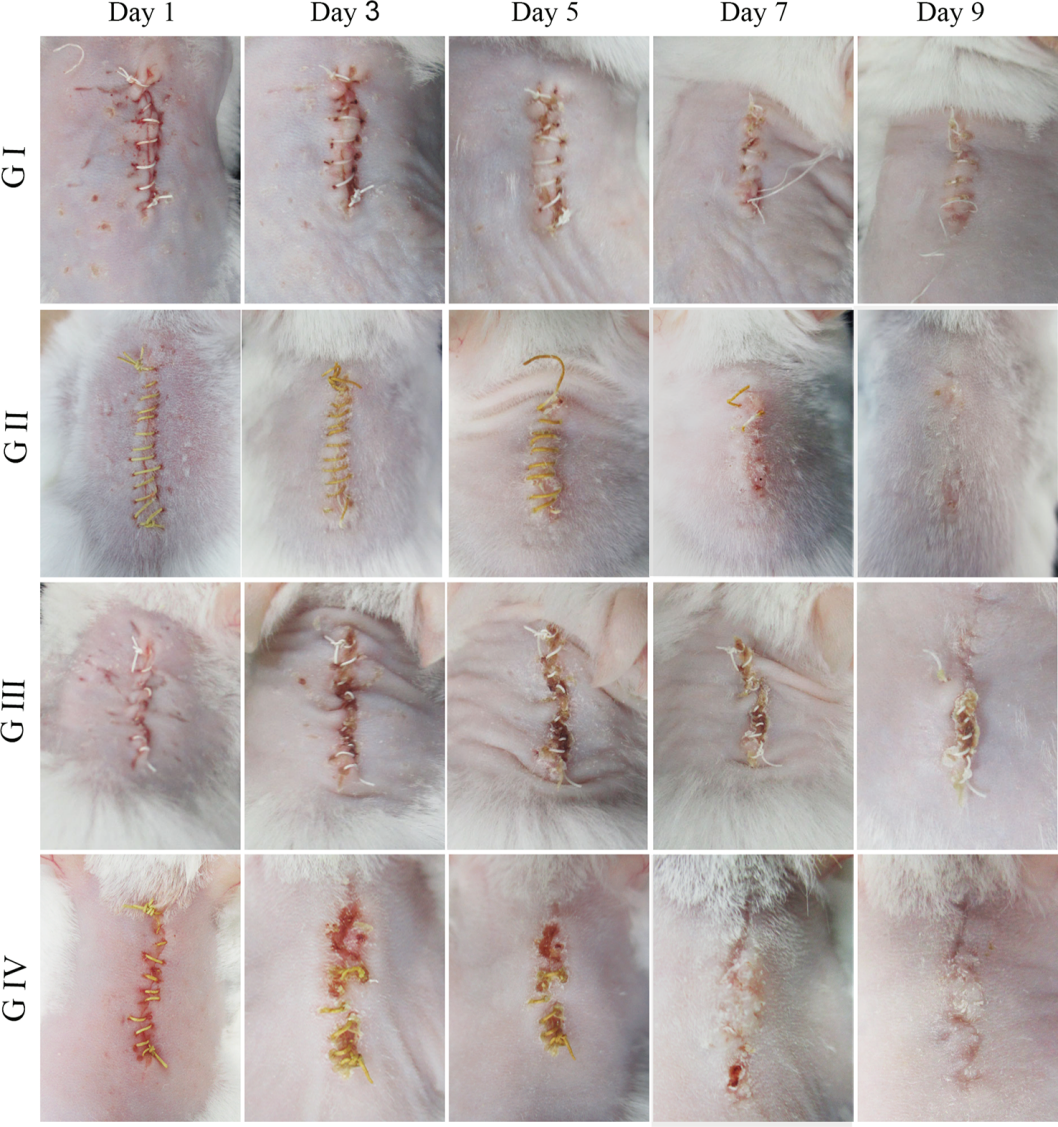
Mouse skin suture–wound model. Representative photos from four experimental subgroups at different times postoperatively show the wound healing process.

The wound healing capacity of the suture was evaluated using a mouse suture–wound model. **Figure 7** shows the wound healing process of the four groups on days 1, 3, 5, 7, and 9 after the operation. On day 1, there was no distinct difference in wound condition among the four groups and no abnormality was observed in each group. In contrast, the skin around the wounds was red and swelling on day 3, indicating that the wounds were inflamed. In particular, the wounds with purulent exudate in G III and G IV were symptomatic of pyogenic infection that was a specific inflammatory caused by a bacterial infection. On day 5, it was shown that the wounds in G III and G IV have severe edema with pus scab, and the involution of these two groups was not good. However, the wounds in G IV were going to heal on day 7, and the ones in G III were still covered with pus scabs and some necrosis tissue. On day 9, the wounds in G III with scab and necrotic tissue remaining had no tendency to heal, while the other three groups have reached clinical healing. Taken together, the above results indicated that the berberine-containing suture promoted wound healing in the presence of *S. aureus* treatment condition.

**Figure 8:**
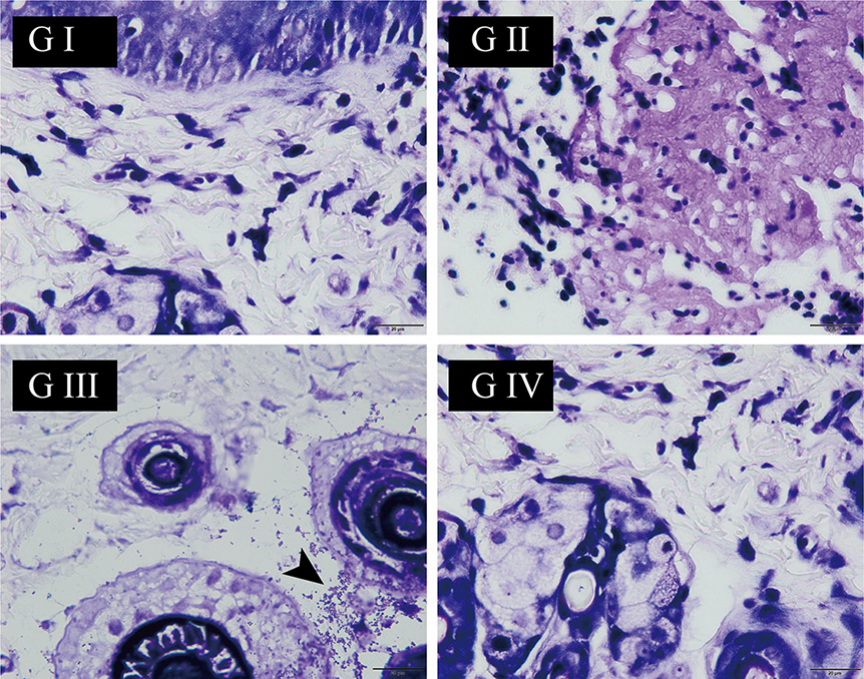
The wound skin tissue was stained with Gram’s crystal violet solution at the end of the experiment on day 9. The *black arrow* points to *Staphylococcus aureus* (the scale is 20 µm).

The antibacterial activity of the suture was assessed by Gram staining. As shown in **Figure 8**, for G I and G II, it is normal to have no bacteria because they are not treated with *S. aureus*. On the contrary, the presence of bacteria was observed in G III (black arrow). Distinctively, G IV had no bacteria in spite of treatment with *S. aureus*, suggesting that the berberine-containing suture had antimicrobial activity.

#### Postoperative Thermoregulation

**Figure 9:**
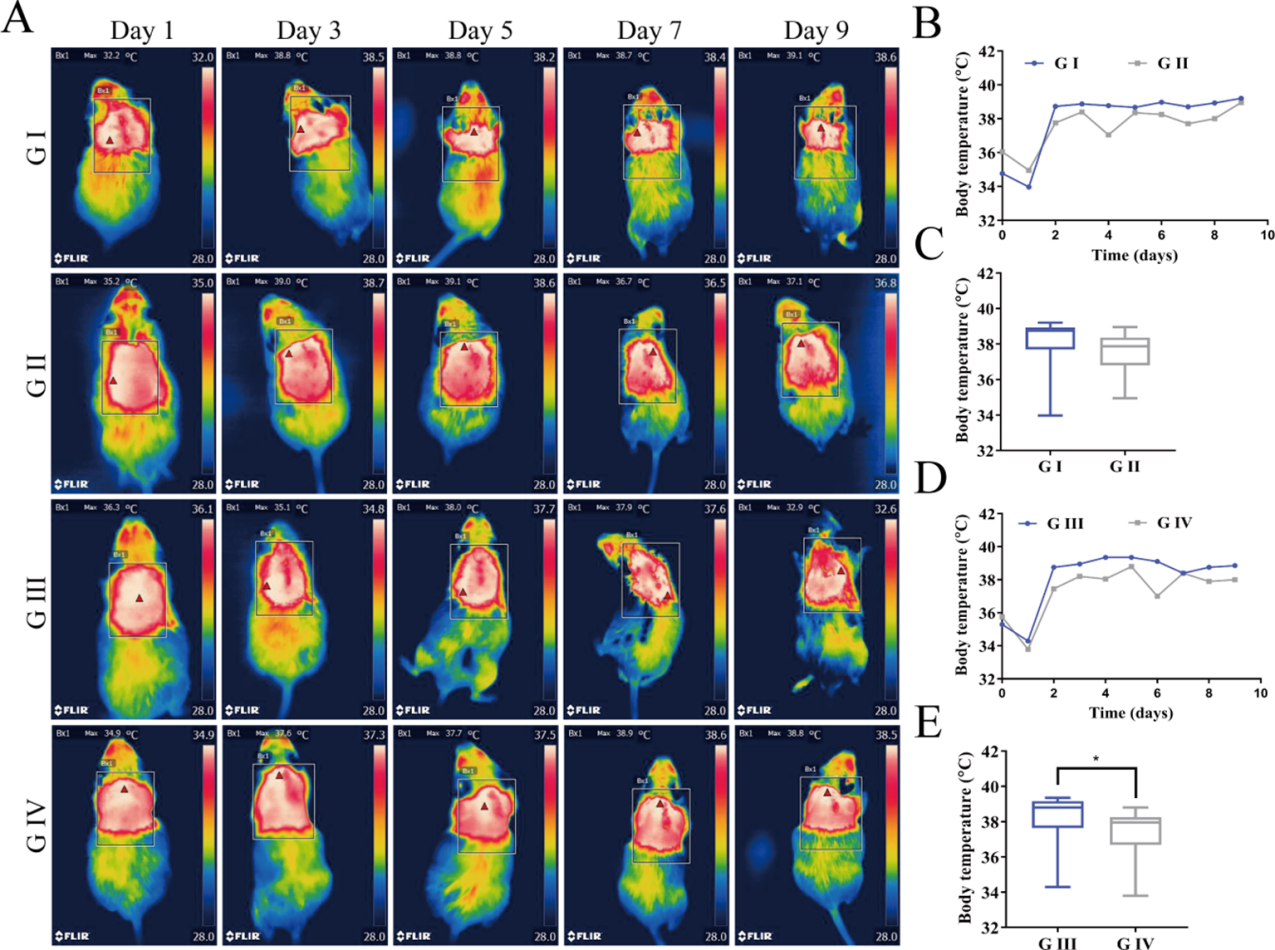
**(A)** Infrared thermal images of the wounds for mice. **(B)** Time courses of the wound temperatures for G I and G II. **(C)** Comparison of the recordings of the temperature for G I and G II using Kruskal–Wallis test. **(D)** Time courses of the wound temperatures for G III and G IV. **(E)** Comparison of the recordings of the temperature for G III and G IV using Kruskal–Wallis test. (*P < 0.05).

The wound temperature was detected by infrared thermal imaging in order to identify if the berberine-containing suture could decrease the temperature of the wound since the signs of inflammation and infection are an increase of temperature. Infrared thermal images and the time courses of the wound temperatures are shown in **Figure 9**. In all groups, the wound temperatures climbed sharply on the second day after the operation (**Figures 9A, B, D**) and then fluctuated around 38°C, indicating that the inflammation and infection induced the increase of the wound temperature. The recordings of the temperature were analyzed by Kruskal–Wallis test, and the results showed that there was no statistical difference in the wound temperature between G I and G II (**Figure 9C**), while the one in G IV was significantly lower than that in G III (**Figure 9E**), suggesting that the berberine-containing suture was capable of anti-inflammation and anti-infection, and thus decrease of the wound temperature. Our results are consistent with the studies of Chu et al. (Chu et al., 2014; Neag et al., 2018), who had demonstrated that berberine possesses the potential to relieve fever as an antipyretic agent.

#### H&E Staining

**Figure 10:**
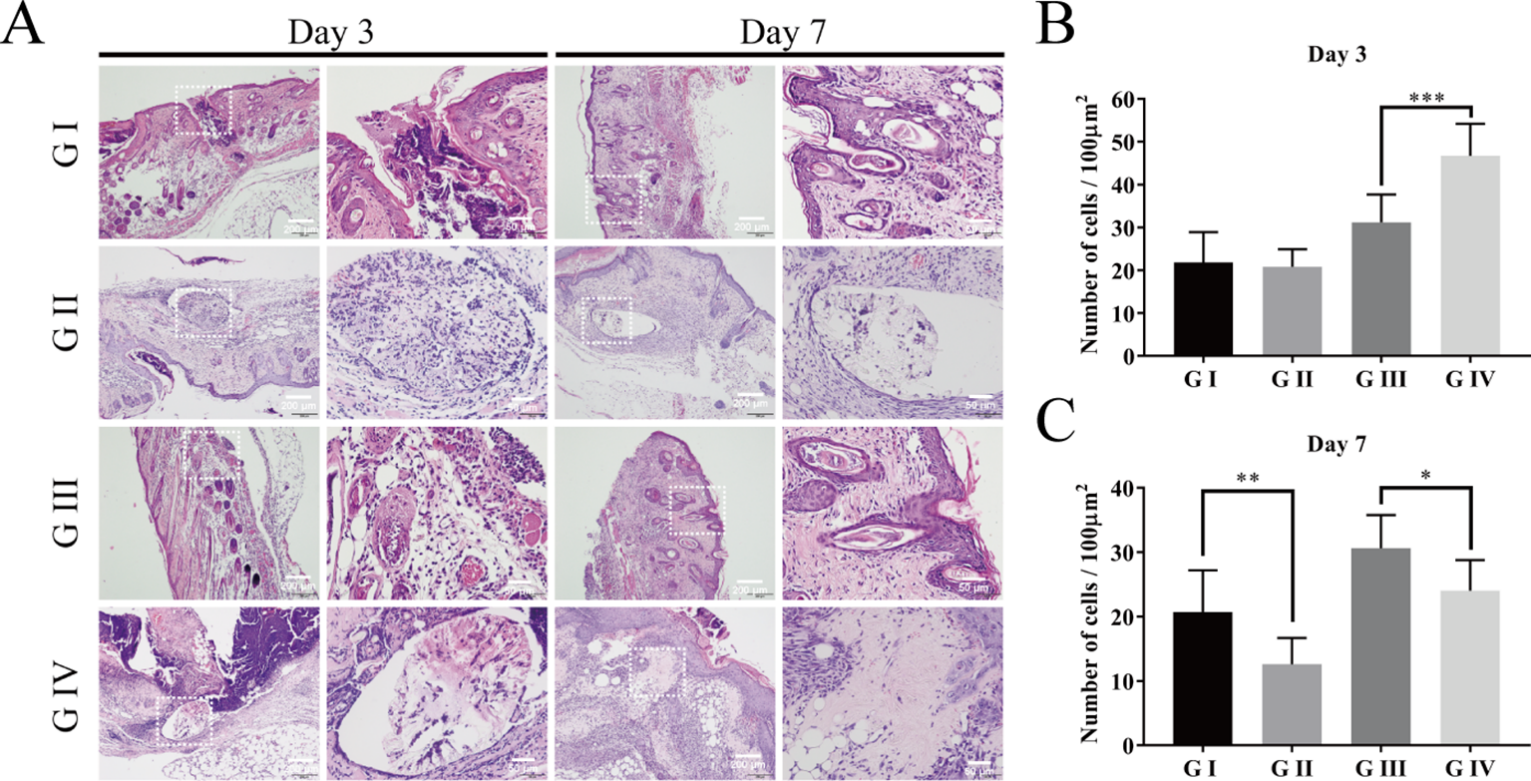
Hematoxylin–eosin staining of the wound skin tissue. **(A)** HE staining on day 3 and day 7; Cumulative percentage of neutrophils in random regions of interest by ImageJ software on day 3 **(B)** and day 7 **(C)**. The scale bar is shown in the figure (one-way ANOVA, mean ± SD, *n* = 6) (*P < 0.05, **P < 0.001, ***P < 0.001).

H&E staining was examined to assess the inflammatory cell response in the wound of the mice (Huang et al., 2019b). On the third day postoperatively, inflammatory cells are concentrated in the skin wound in all groups (**Figure 10A**). The severity of the inflammation was estimated by the number of neutrophils (Fan et al., 2018). As shown in **Figure 10B**, the numbers of neutrophils in G III and IV are significantly more than those in G I and G II, indicating that infection with *S. aureus* can lead to severe inflammation in the wound of the mice. When comparing G III and G IV, it was shown that the neutrophils in G IV were much more than those in G III, which suggested that berberine-containing suture could enhance the recruitment of neutrophils around the suture in the incipient process of inflammation.

When the time prolonged to day 7, both the numbers of the neutrophils in G II and G IV were significantly decreased compared to those on day 3, respectively, indicating that the inflammation is significantly suppressed on day 7. Moreover, the numbers of neutrophils in G II and G IV were also significantly less than those in G I and G III on day 7, respectively (**Figure 10C**). These tendencies proved the anti-inflammatory effect of the berberine-containing suture.

Although traditional Chinese medicine has been using berberine-containing herbs to treat inflammatory bowel disease for thousands of years, and many previous studies have revealed the anti-inflammatory properties of berberine, barely any tests have been performed for the purpose of verifying the same effect in skin tissue (Hasday et al., 2000; Küpeli et al., 2002; Doyle and Schortgen, 2016). Our study has not only demonstrated the same pharmaceutical properties in skin tissue but also showed time specificity.

#### Immunohistochemistry Staining

**Figure 11:**
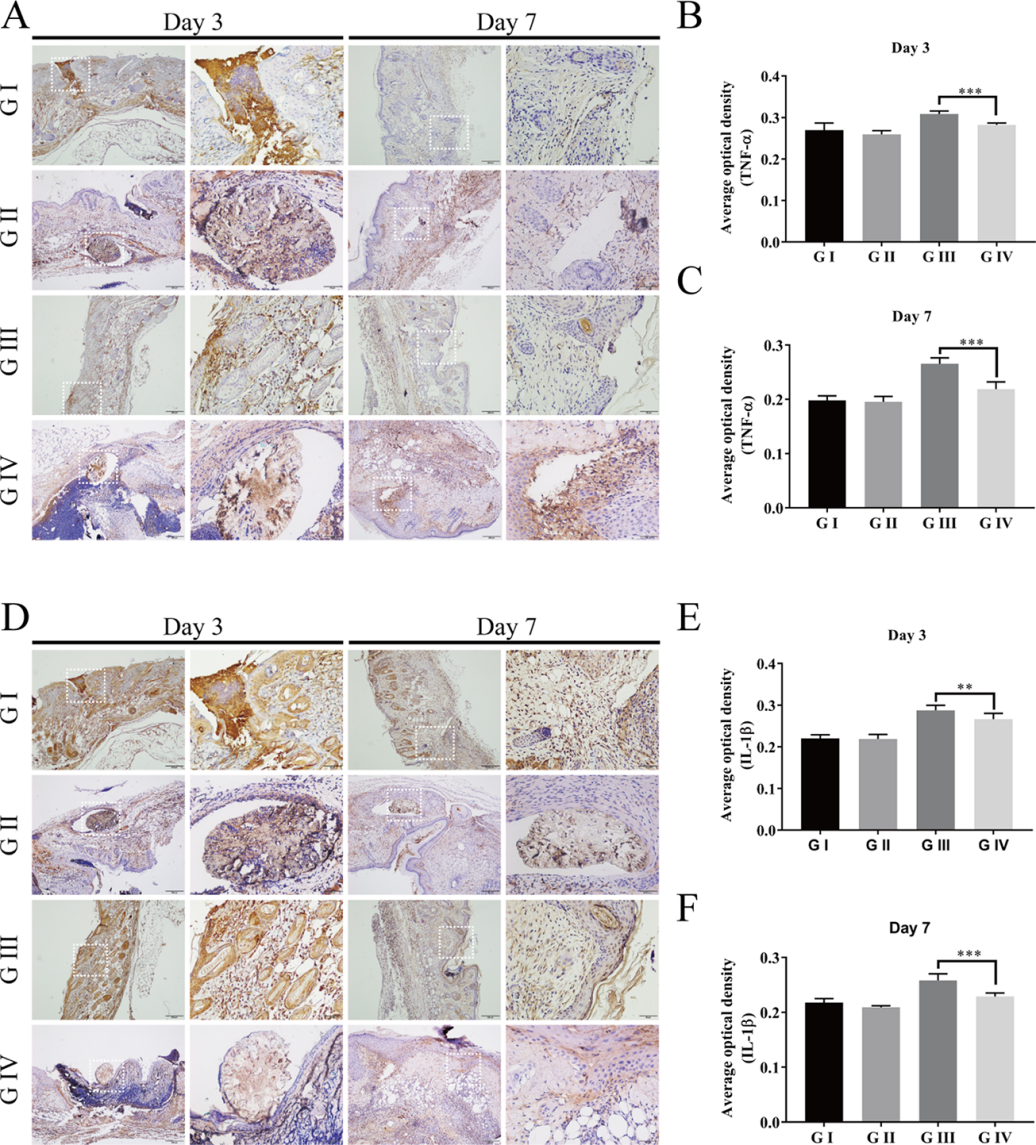
Immunohistochemistry of the wound skin tissue. The immunohistochemical staining of pro-inflammatory cytokines TNF-α **(A)** and IL-1β **(D)** of the wound skin tissue. Average optical density of TNF-α on day 3 **(B)** and day 7 **(C)**; Average optical density of IL-1β on day 3 **(E)** and day 7 **(F)**. The scale bar is shown in the figure (one-way ANOVA, mean ± SD, *n* = 6) (**P < 0.01, ***P < 0.001).

To further investigate the anti-inflammation effects of the berberine-containing suture, pro-inflammatory cytokines, such as TNF-α and IL-1β, were determined to assess the inflammation by immunohistochemical staining. As shown in **Figures 11A–F**, both TNF-α and IL-1β in G III and G IV exhibited higher levels than those in G I and G II on both days 3 and 7, confirming that the infection with *S. aureus* resulted in severe inflammation, as evidenced by H&E staining. More importantly, both cytokines in G IV were always lower than those in G III on both days 3 and 7, indicating that the berberine-containing suture is capable of reducing pro-inflammatory cytokines in the course of inflammation, which is consistent with its anti-inflammation effects.

#### Mechanism Discussion

Based on the results from the animal study using mouse skin suture–wound model, the berberine-containing suture exhibited promotion of wound healing, antibacterial activity, decrease of wound temperature, as well as anti-inflammatory effect, all of which seem to be related to the bioactivities of berberine that was incorporated in the suture since berberine is hydrophilic and released from the suture in a sustained manner. To explain these beneficial effects of the berberine-containing suture in the mouse model, a mechanism was proposed as follows based on literature and our results.

**Figure 12:**
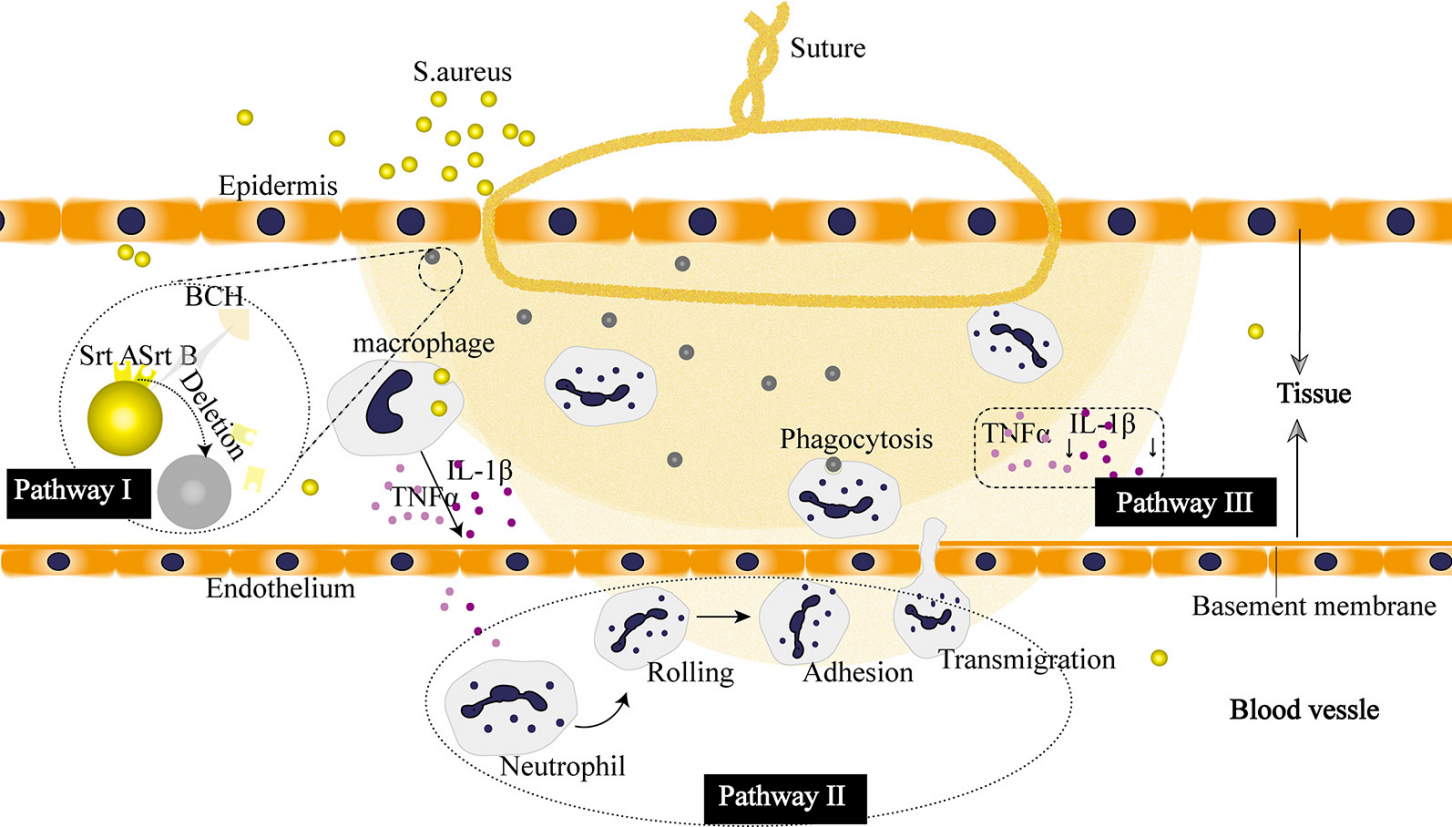
Proposed pharmacological mechanism of the berberine released from the suture. **I** Berberine inhibits virulence and infection potential of *Staphylococcus aureus* through restraining the activities of sortase **A** (*SrtA*) and sortase **B** (*SrtB*). **II** Berberine enhances the recruitment of neutrophils. **III** Berberine plays an anti-inflammatory role by downregulating the expression of cytokines.

First, the potential benefit of the berberine-containing suture in wound healing could come from the widely known antibacterial activity of the incorporated berberine. Berberine is a positively charged compound, endowing itself a property to interact directly with bacterial cell wall components, lipopolysaccharide, and cell surface proteins. In particular, Oh et al. revealed an important antibacterial mechanism that berberine possessed strong inhibition activity on sortase enzymes, leading to a remarkable reduction in the virulence and infection ability of *S. aureus* (Oh et al., 2006). The sortase enzyme is a surface protein transpeptidase relevant to anchoring the surface proteins in the cell wall of peptidoglycan in Gram-positive bacteria. For *S. aureus*, the sortase enzyme plays a pivotal role in the adhesion to the host, making them accomplices to bacterial infection. Berberine acts on *S. aureus* by inhibiting the adhesion process through deletion of two sortase enzyme isoforms, sortase A (SrtA) and sortase B (SrtB), to restrain the activities of the sortases, and thus exhibiting antibacterial activity (**Figure 12I**).

Second, our results showed that the berberine-containing suture enhanced the recruitment of neutrophils in the early stage of inflammation (**Figure 10**). The schematic diagram is shown in **Figure 12II**. In order to find out the reasons for this phenomenon, we need to see the essence of what happened. As an adaptive response to noxious conditions including infection and injury, inflammation is crucial to restoring homeostasis, even though it can affect normal physiology, resulting in a transient decline in tissue function. Inflammation is a double-edged sword for health. It is helpful for the body in reasonable amounts (Chang et al., 2018). Meanwhile, it is easy to become detrimental when of excessive duration or high intensity on account of its tissue-damaging potential (Habtemariam, 2016). Thus, it is essential for inflammation to be orchestrated in the right way. The berberine-containing suture in our study just has the ability to regulate inflammation, e.g., it can enhance the inflammation in the early stage and then suppress inflammation subsequently as well.

Besides, the expression of cytokines is significantly inhibited throughout the later stages of the inflammatory response by the berberine-containing suture (**Figure 12III**). Pro-inflammatory cytokines, such as TNF-α and IL-1β, play an important role in activating inflammation. They are fundamental in the incipient stages of inflammation. Nevertheless, it is harmful if the cytokines keep working throughout the course of inflammation. Blocking the cascade amplification of cytokines in time can reduce the damage to the tissues caused by inflammation. Lee et al. also observed that berberine was capable of suppressing inflammatory agent-induced TNF-α and IL-1β production in lung cells (Lee et al., 2007). Furthermore, their results showed that the suppression effect of berberine resulted from the inhibition of inhibitory κB-α phosphorylation and degradation.

Overall, it is interesting that berberine-containing suture is antibacterial and anti-inflammatory, plus its shape memory effect, all of which will make it attractive to extend the use of the suture in a wide range of clinical applications.

## Conclusions

In summary, we report a novel berberine-containing surgical suture that possesses both antibacterial activity and shape memory effect to address clinical issues such as SSIs and difficulty in handling in minimally invasive surgery. The sutures were fabricated by a facile scalable one-step wet-spinning strategy, in which natural berberine was incorporated directly into the spinning solution of shape memory polyurethane with a *T*_m_ slightly higher than body temperature, and then were comprehensively characterized in terms of their transition temperature, morphology, water contact angles, mechanical properties, *in vitro* shape memory effect, drug release, antibacterial activity, and compatibility. The optimized fiber was further evaluated by animal studies, and the results showed that the fiber was capable of shape memory, antibacterial activity, and anti-inflammation, and thus promote wound healing. In addition, the mechanism of antibacterial activity and anti-inflammatory effect was discussed.

## Data Availability Statement

All datasets generated for this study are included in the article/**Supplementary Material**.

## Ethics Statement

The animal study was reviewed and approved by the Animal Ethical Committee of the West China Hospital of Sichuan University.

## Author Contributions

W-CZ: animal studies, writing the article, and results discussion. P-FT: synthesis of shape memory polyurethane, preparation of shape memory fibers, and statistical analysis. X-HC and HL performed the other experiments. LT and MT were responsible for conceptualizing and revising the manuscript. CY and YC were involved in conceptualizing and proofreading. All authors gave their final approval for the submission of the manuscript.

## Funding

This work was sponsored by the National Natural Science Foundation of China (no. 51803128), Opening Project of Key Laboratory of Leather Chemistry and Engineering (Sichuan University), Ministry of Education (grant no. 20826041C4159), Sichuan Science and Technology Programs (grant no. 2017SZYZF00009, no. 19YJ0126), and Strategic Project of Lu Zhou Science & Technology Bureau (grant no. 2017CDLZ-S01).

## Conflict of Interest

The authors declare that the research was conducted in the absence of any commercial or financial relationships that could be construed as a potential conflict of interest.

## Supplementary Material

The Supplementary Material for this article can be found online at: https://www.frontiersin.org/articles/10.3389/fphar.2019.01506/full#supplementary-material

## References

[B1] Avila-Carrasco L. Majano P. Sánchez-Toméro J. A. Selgas R. López-Cabrera M. (2019). Natural plants compounds as modulators of Epithelial-to-Mesenchymal transition. Front. Pharmacol. 10, 715. 10.3389/fphar.2019.00715 31417401 PMC6682706

[B2] Bernard M. Jubeli E. Bakar J. Tortolano L. Saunier J. (2017). Biocompatibility assessment of cyclic olefin copolymers: impact of two additives on cytotoxicity, oxidative stress, inflammatory reactions, and hemocompatibility. J. BioMed. Mater Res. 105, 3333–3349. 10.1002/jbm.a.36199 28875577

[B3] Byrne M. Aly A. (2019). The surgical suture. Aesthetic Surg. J. 39, S67–S72. 10.1093/asj/sjz036 30869751

[B4] Chang J. B. Lane M. E. Yang M. Heinrich M. (2018). Disentangling the complexity of a Hexa-Herbal chinese medicine used for inflammatory skin conditions—predicting the active components by combining LC-MS-Based metabolite profiles and *in vitro* pharmacology. Front. Pharmacol. 9, 1091. 10.3389/fphar.2018.01091 30344490 PMC6182074

[B5] Chu M. Ding R. Chu Z. Y. Zhang M. B. Liu X. Y. (2014). Role of berberine in anti-bacterial as a high-affinity LPS antagonist binding to TLR4/MD-2 receptor. BMC Complement Altern. Med. 14, 89. 10.1186/1472-6882-14-89 24602493 PMC3946165

[B6] Ding J. Zhang J. Li J. Li D. Xiao C. (2019). Electrospun polymer biomaterials. Prog. Polymer Sci. 90, 1–34. 10.1016/j.progpolymsci.2019.01.002

[B7] Doyle J. F. Schortgen F. (2016). Should we treat pyrexia? And how do we do it? *Critical Care* 20. 10.1186/s13054-016-1467-2 PMC5047044 27716372

[B8] Fan H. Xie Z. Lu Z. Tan Z. Bi Y. (2018). Anti-inflammatory and immune response regulation of Si-Ni-San in 2,4-dinitrochlorobenzene-induced atopic dermatitis-like skin dysfunction. J. Ethnopharmacology 222, 1–10. 10.1016/j.jep.2018.04.032 29698775

[B9] Feng X. Li J. Zhang X. Liu T. Ding J. (2019). Electrospun polymer micro/nanofibers as pharmaceutical repositories for healthcare. J. Controlled Release 302, 19–41. 10.1016/j.jconrel.2019.03.020 30922946

[B10] Habtemariam S. (2016). Berberine and inflammatory bowel disease: a concise review. Pharmacol. Res. 113, 592–599. 10.1016/j.phrs.2016.09.041 27697643

[B11] Hahn B. L. Onunkwo C. C. Watts C. J. Sohnle P. G. (2009). Systemic dissemination and cutaneous damage in a mouse model of staphylococcal skin infections. Microb. Pathog. 47, 16–23. 10.1016/j.micpath.2009.04.007 19397991 PMC2831771

[B12] Hasday J. D. Fairchild K. D. Shanholtz C. (2000). The role of fever in the infected host (France: Elsevier SAS). 10.1016/s1286-4579(00)01337-x 11165933

[B13] Huang J. Chen L. Gu Z. Wu J. (2019a). Red Jujube-Incorporated gelatin methacryloyl (GelMA) hydrogels with Anti-Oxidation and immunoregulation activity for wound healing. J. BioMed. Nanotechnol. 15, 1357–1370. 10.1166/jbn.20192815 31196343

[B14] Huang J. Chen L. Yuan Q. Gu Z. Wu J. (2019b). Tofu-Based hybrid hydrogels with antioxidant and low immunogenicity activity for enhanced wound healing. J. BioMed. Nanotechnol. 15, 1371–1383. 10.1166/jbn.20192814 31196344

[B15] Joseph B. George A. Gopi S. Kalarikkal N. Thomas S. (2017). Polymer sutures for simultaneous wound healing and drug delivery – a review. Int. J. Pharmaceutics 524, 454–466. 10.1016/j.ijpharm.2017.03.041 28385650

[B16] Küpeli E. Koşar M. Yeşilada E. Başer K. H. C. (2002). A comparative study on the anti-inflammatory, antinociceptive and antipyretic effects of isoquinoline alkaloids from the roots of Turkish Berberis species. Life Sci. 72, 645–657. 10.1016/S0024-3205(02)02200-2 12467905

[B17] Lee C. Chen J. Hsiang C. Wu S. Wu H. (2007). Berberine suppresses inflammatory agents-induced interleukin-1β and tumor necrosis factor-α productions *via the* inhibition of IκB degradation in human lung cells. Pharmacol. Res. 56, 193–201. 10.1016/j.phrs.2007.06.003 17681786

[B18] Lendlein A. Langer R. (2002). Biodegradable, elastic shape-memory polymers for potential biomedical applications. Science 296, 1673–1676. 10.1126/science.1066102 11976407

[B19] Mebert A. M. Alvarez G. S. Peroni R. Illoul C. Hélary C. (2018). Collagen-silica nanocomposites as dermal dressings preventing infection *in vivo* . Mater. Sci. Eng.: C. 93, 170–177. 10.1016/j.msec.2018.07.078 30274049

[B20] Mullick Chowdhury S. Lalwani G. Zhang K. Yang J. Y. Neville K. (2013). Cell specific cytotoxicity and uptake of graphene nanoribbons. Biomaterials 34, 283–293. 10.1016/j.biomaterials.2012.09.057 23072942 PMC3489471

[B21] Nakagawa S. Matsumoto M. Katayama Y. Oguma R. Wakabayashi S. (2017). Staphylococcus aureus virulent PSMα peptides induce keratinocyte alarmin release to orchestrate IL-17-Dependent skin inflammation. Cell Host Microbe 22, 667–677. 10.1016/j.chom.2017.10.008 29120744 PMC5728420

[B22] Neag M. A. Mocan A. Echeverría J. Pop R. M. Bocsan C. I. (2018). Berberine: Botanical occurrence, traditional uses, extraction methods, and relevance in cardiovascular, metabolic, hepatic, and renal disorders. Front. Pharmacol. 9, 557. 10.3389/fphar.2018.00557 30186157 PMC6111450

[B23] Oh K. Oh M. Kim J. Shin D. Shin J. (2006). Inhibition of sortase-mediated Staphylococcus aureus adhesion to fibronectin *via* fibronectin-binding protein by sortase inhibitors. Appl. Microbiol. Biotechnol. 70, 102–106. 10.1007/s00253-005-0040-8 16010573

[B24] Shao K. Han B. Gao J. Jiang Z. Liu W. (2016). Fabrication and feasibility study of an absorbable diacetyl chitin surgical suture for wound healing. J. BioMed. Mater Res. B. Appl. Biomater. 104, 116–125. 10.1002/jbm.b.33307 25677094

[B25] Tang J. Zhao R. Yin X. Wen Y. Shi Y. (2019). Programmable release of berberine chloride hydrate from shape memory fibers prepared from core-sheath wet-spinning technology. J. Biomed. Nanotechnol. 15, 1432. 10.1166/jbn.2019.2784 31196348

[B26] Wang S. Yan C. Zhang X. Shi D. Chi L. (2018). Antimicrobial peptide modification enhances the gene delivery and bactericidal efficiency of gold nanoparticles for accelerating diabetic wound healing. Biomater. Sci. 6, 2757–2772. 10.1039/C8BM00807H 30187036

[B27] Zhao W. Liu L. Zhang F. Leng J. Liu Y. (2019). Shape memory polymers and their composites in biomedical applications. Mater. Sci. Eng. C. 97, 864–883. 10.1016/j.msec.2018.12.054 30678978

