# Supplementary material for: Berberine-Incorporated Shape Memory Fiber Applied as a Novel Surgical Suture
Source: Front Pharmacol. 2020 Jan 9;10:1506. doi: 10.3389/fphar.2019.01506 (PMC6962190; doi:10.3389/fphar.2019.01506)
Supplement: Supplementary file 1 [file Table_1.docx]

Supplementary Material

# Supplementary Tables

Table S1. The spinning parameters of fibers

| Fibers | Syringe speed  mm/min | Drawing roller speed  m/min | Primary drawroll speed  m/min | Secondary stretch roll speed  m/min | Traverse speed  times/min | Roll speed  m/min |
| --- | --- | --- | --- | --- | --- | --- |
| BP-0 | 1.0 | 1.5 | 1.8 | 2.0 | 1 | 2.0 |
| BP-1 | 1.0 | 1.5 | 1.8 | 2.0 | 1 | 2.0 |
| BP-2 | 1.0 | 1.5 | 1.8 | 2.0 | 1 | 2.0 |

Table S2. The number of colonies after co-culture.

| Colony count (CFU/mL) | Blank | BP-0 | BP-1 | BP-2 |
| --- | --- | --- | --- | --- |
| E. coli | 1.3×10^6^ | 2.3×10^6^ | 0 | 0 |
| S. aureus | 4.5×10^5^ | 6×10^5^ | 0 | 0 |
